# Virus Pathogens in Australian Vineyards with an Emphasis on Shiraz Disease †

**DOI:** 10.3390/v12080818

**Published:** 2020-07-28

**Authors:** Qi Wu, Nuredin Habili, Fiona Constable, Maher Al Rwahnih, Darius E. Goszczynski, Yeniu Wang, Vinay Pagay

**Affiliations:** 1 School of Agriculture, Food & Wine, University of Adelaide, Waite Precinct, PMB 1, Glen Osmond, Adelaide 5064, South Australia, Australia; qi.wu@adelaide.edu.au (Q.W.); yeniu.wang@adelaide.edu.au (Y.W.); 2 The Australian Wine Research Institute, PO Box 197, Glen Osmond, Adelaide 5064, South Australia, Australia; nuredin.habili@awri.com.au; 3 Agriculture Victoria Research, Department of Economic Development, Jobs, Transport and Resources, AgriBio, Bundoora, Melbourne 3083, Victoria, Australia; fiona.constable@agriculture.vic.gov.au; 4 Department of Plant Pathology, University of California, Davis, CA 95616, USA; malrwahnih@ucdavis.edu; 5 Plant Protection Research Institute, Agricultural Research Council, Private Bag X134, Pretoria 0001, South Africa; goszczynskid@arc.agric.za

**Keywords:** grapevine, high throughput sequencing, vectors, rogueing, leafroll disease, scale insects, mealybugs

## Abstract

Grapevine viruses are found throughout the viticultural world and have detrimental effects on vine productivity and grape and wine quality. This report provides a comprehensive and up-to-date review on grapevine viruses in Australia with a focus on “Shiraz Disease” (SD) and its two major associated viruses, grapevine virus A (GVA) and grapevine leafroll-associated virus 3 (GLRaV-3). Sensitive grapevine cultivars like Shiraz infected with GVA alone or with a co-infection of a leafroll virus, primarily GLRaV-3, show symptoms of SD leading to significant yield and quality reductions in Australia and in South Africa. Symptom descriptors for SD will be outlined and a phylogenetic tree will be presented indicating the SD-associated isolates of GVA in both countries belong to the same clade. Virus transmission, which occurs through infected propagation material, grafting, and naturally vectored by mealybugs and scale insects, will be discussed. Laboratory and field-based indexing will also be discussed along with management strategies including rogueing and replanting certified stock that decrease the incidence and spread of SD. Finally, we present several cases of SD incidence in South Australian vineyards and their effects on vine productivity. We conclude by offering strategies for virus detection and management that can be adopted by viticulturists. Novel technologies such as high throughput sequencing and remote sensing for virus detection will be outlined.

## 1. Introduction

*Vitis vinifera,* cv. Shiraz (syn. Syrah) is the most popular cultivar in Australia. In 2018, of the total of 135,133 ha under cultivation, Shiraz accounted for 39,893 ha (approximately 30% of total winegrape acreage in Australia) making it the most widely planted winegrape cultivar in Australia (www.wineaustralia.com/market-insights/australian-wine-sector-at-a-glance). Of 86 viruses detected in the grapevine to date [1], 35 have been reported to have negative effects on vine performance, especially in red-berried cultivars [2]. Grapevine virus A (GVA), grapevine virus B (GVB), grapevine leafroll associated viruses (GLRaV-1, -2, -3, and -4), grapevine rupestris stem pitting associated virus (GRSPaV) and grapevine fleck virus (GFkV) have been found in Australian vineyards historically [3]. The most recent viruses reported from Australia are grapevine Pinot gris virus (GPGV) and grapevine rupestris vein feathering virus [4]. Grapevine fanleaf virus has been eradicated and it is now listed as quarantined [5].

Sensitivity of different grapevine cultivars to viruses is variable [6]. Among the red-berried cultivars, Shiraz is highly sensitive to infections by GVA; symptoms associated with GVA infections include retarded shoot growth, decreased sugar accumulation in the berries and, in some cases, even vine mortality [7,8]. The disease is called Shiraz Disease (SD), which is one of the most debilitating diseases of Shiraz in Australia and South Africa [9]. This disease was first reported in 1985 from South Africa without knowledge of its associated virus [10]. In 2003, it was reported that GVA was associated with the disease in both South Africa and Australia [11,12]. The first GVA infection in Australia was detected by our group in 1997 in symptomatic Shiraz vines in the Clare Valley, South Australia. SD is associated with either infection by GVA alone, or co-infection of GVA with one or more of leafroll viruses such as GLRaV-3, GLRaV-4 strain 9, or GLRaV-1 in sensitive red-berried cultivars like, Shiraz, Merlot, Malbec, and Sumoll. The virus associated disease occurs only in South Africa and Australia, and it appears to be different from Syrah decline in the USA and France, which may be a genetic disease [13,14].

In this review, we will refer to viruses affecting grapevines in Australia with an emphasis on those associated with SD. Information on the nature of the associated viruses, symptom expression, genomics, and detection will be provided. We then review the effects of SD on grapevine growth, physiological function, and fruit and wine composition. We conclude by sharing some recent observations of SD-affected vineyards in South Australia and proposing strategies for disease control.

## 2. Grapevine Viruses in Australian Vineyards

Viruses are widespread in vineyards worldwide. To date, few viruses have been detected in grapevines in Australia compared to globally (Table 1); this is partly attributed to strict biosecurity measures that have prevented the introduction of infected material into Australia as well as the lack of more efficient vectors [15,16,17]. The selection of material from productive clones and cultivars, virus-testing, in vitro virus eradication, and established certification programs have assisted in preventing some of the more serious diseases and pathogens of grapevine entering or dispersing within Australia. Nevertheless, some serious virus associated diseases, including leafroll disease and diseases of the rugose wood complex, occur and spread in Australian vineyards (Table 1).

## 3. Virus Transmission in Vineyards

Many of the commonly occurring grapevine viruses are specific to *Vitis* sp. so the likelihood of the virus infection spreading to or from another perennial or herbaceous plant is low. Spread of the major viruses, particularly GVA and GLRaV-1 and -3, in Australian vineyards occurs primarily through infected propagation material; there is no evidence to date of mechanical transmission, including pruning. In established vineyards, vector transmission is thought to be the dominant mode of virus transmission between vines.

### 3.1. Primary Transmission

Primary transmission is the introduction of a virus infection into a crop. This type of transmission often occurs in vineyards following establishment using propagation material sourced from infected mother vines, but it could also occur when a viruliferous vector is introduced to a vineyard from another area. Over centuries, primary transmission of viruses has inadvertently been practiced by humans using infected cuttings for propagating own-rooted and grafted plants and top working. Top-working—grafting by either chip bud, T-bud or cleft methods—new cultivars onto an existing virus-infected grapevine is a common practice by vignerons in Australia [12,18]. However, this practice is risky if the virus status of a grapevine to be top-worked is unknown and symptoms are not apparent. One example of this trend is top-working of the popular Australian cultivar Shiraz onto Chardonnay or Riesling. When infected with GVA, these white-berried cultivars do not show typical red-leaf symptoms as observed in red-berried cultivars, but rather appear “clean”, i.e., asymptomatic or having a faint chlorotic appearance on leaves. However, GVA is associated with drastic symptoms in Shiraz, Malbec, and Merlot in Australia following grafting (see below). A random distribution of infected grapevines within a block is often indicative of primary spread into a vineyard and suggests that either infected material was planted randomly across the block or the viruliferous vectors were transported long distance by wind or other carriers.

### 3.2. Secondary Transmission

Once a primary infection is established in a vineyard, secondary infections or spread within a block can occur via vectors such as mealybugs and scale insects [19,20,21,22,23]. When secondary virus spread occurs, the pattern of infected vines is aggregated [24], because viruliferous mealybug nymphs (crawlers) transmit the virus to vines located at close proximity to the primary infection. Adjacent vines within a row and neighbouring vines in adjacent rows may become infected forming a cluster (“hot spot”) around the primary infection within a vineyard. Gradients of infection can be observed when transmission occurs from infected vines located at the edge of vineyards [25]. Over the course of several seasons, entire vineyards can become infected. The rate of secondary spread is likely correlated with the abundance of vectors. For example, in a French vineyard, an increase in leafroll disease incidence from 5% to 86% over eight years was linked to a 74% incidence of *Phenacoccus aceris* during the same period [26]. A study in an Australian Pinot noir vineyard showed that the incidence of GLRaV-3 increased from 23% to 52% over a 10 year period, with no change in incidence until the last three years suggesting a change in ecology of the virus vectors [27]. The same study also found that the rate of spread was one-third of that observed in a NZ Cabernet Sauvignon vineyard; this might be attributed to differences in vector species and population dynamics between the two regions. Therefore, it is critical to understand vector populations and ecology so that efficient management strategies can be developed to control secondary virus spread.

The spread of phloem-limited grapevine viruses such as GVA and GLRaV-3 is mediated by mealybugs (Family: *Pseudococcidae*) and soft scales (Family: *Coccidae*) [25,28]. Co-infections of GVA with GLRaV-1, GLRaV-3, or GLRaV-4 strain 9 have been observed [7], and these viruses can be transmitted simultaneously. In adult mealybugs, *Pseudococcus viburni* (Signoret), isolated from the cultivar Shiraz infected with SD, both GLRaV-3 and GVA were detected, and further work is required to understand the role of this vector and other vectors in SD spread. Table 2 lists the mealybug vectors, and Table 3 lists the scale insect vectors of grapevine viruses, including those that occur in Australia.

Transmission of grapevine viruses by all mealybug and scale species is thought to be in a non-circulative, semipersistent manner: viruses may be acquired, but they do not replicate in the insect. Instead viruses are retained in the foregut and transmitted after hours or days [44,45]. Research suggests that there is no virus-vector specificity, and multiple mealybug species can transmit one virus species (Table 2). Conversely, a single mealybug species can transmit multiple GLRaV species and some vitiviruses [46]. GLRaV-3 appears to be transmitted more efficiently by mealybugs than other viruses [25,47]. A recent study found that virus transmission by mealybugs was 22% more efficient for GLRaV-3 than GVA, and that GVA transmission was enhanced in the presence of GLRaV-3 [47]. First and second instars nymphs (crawlers) are more efficient at spreading the leafroll viruses and GVA because these viruses are easily dispersed within and between vineyards by crawlers, wind, and mechanical methods [48,49]. Certain mealybug species have been reported to have up to six generations per year [50]. Population size and number of generations of the insect in a single season are key factors influencing virus transmission rates, and high numbers can lead to rapid spread of viruses. Transmission of viruses within or between vineyards can be facilitated by movement of mealybug nymphs on vineyard equipment, ants, humans, and wind [51]. Evidence for the mode of spread can be found from the pattern of infection in the newly infected vineyard: clusters of virus infection may be observed near equipment entry points in the vineyard or along edges next to other infected vineyards. Mealybug crawlers, which are most efficient in virus transmission [52], are very light and can therefore be easily blown by wind from vineyard blocks as far as several kilometers away, depending on humidity and temperature [53]. In wind-borne transmission of the virus, the resulting distribution of associated disease in the vineyard may appear random, or edge effects might be observed [54].

## 4. Symptomatology

The ability to detect virus infections based on symptoms is largely dependent on experience and the specific combination of virus species, grapevine cultivar, and geographical region (e.g., symptoms of GLRaV-3 on Shiraz cultivar in Thailand; N Habili, unpublished). The challenge with symptom-based identification is the fact that several viruses can be present in a host, altering symptom expression. Certain *Vitis vinifera* cultivars may be symptomless or show only minor leaf chlorosis, such as many white-berried cultivars (e.g., Sauvignon Blanc, Chardonnay, and Riesling) as well as *Vitis* rootstocks. The genetic variability of a virus species also influences symptom expression. Multiple genetic variants of specific viruses including GVA [62] and GRSPaV [63,64], both of which are found in Australian vineyards, have been observed to exist in an individual grapevine and are thought to have occurred via grafting, vectors, and/or pollination [63]. Virus infections in grapevines that are symptomatic can be mistaken with certain nutrient deficiencies or phytoplasma-associated diseases such as Australian grapevine yellows and flavescence dorée. Viruses can produce specific symptoms on the vine, and these symptoms sometimes form part of the virus name. Below, we describe the symptoms of SD associated with GVA in conjunction with GLRaV infection [65] with special reference to Australia. Readers are referred to [66] and [6] for a detailed description of grapevine virus symptomatology.

### 4.1. Shiraz Disease

**Figure 1:**
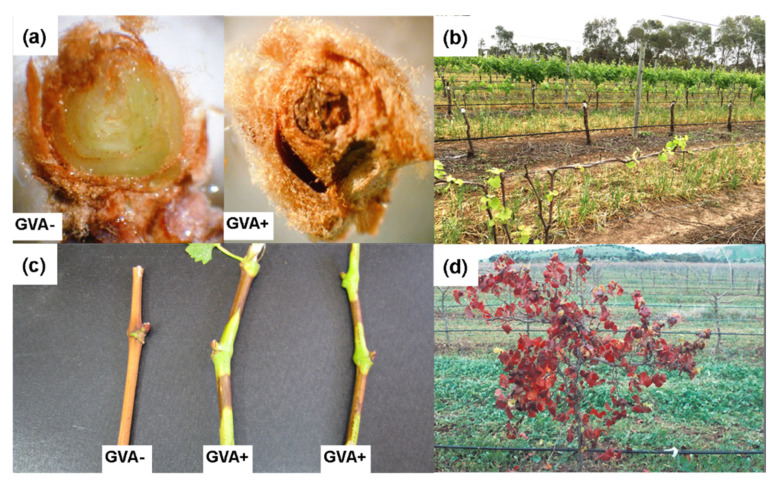
Symptomatology of grapevine virus A (GVA)-associated Shiraz Disease. (**a**) primary bud necrosis shown on right; (**b**) restricted spring growth (front row); (**c**) partial lignification showing islands of green immature canes; (**d**) retention of crimson coloured leaves on canopy in winter.

In GVA-infected *V. vinifera* cv. Shiraz (syn. Syrah), symptoms of primary bud necrosis (PBN) are often observed in buds during the dormant season (Figure 1a). A survey of GVA-positive Shiraz vines in a South Australian vineyard found that 41% of primary buds had PBN, while in GVA-negative Shiraz vines, only 11% had PBN.

Early in the growing season, shoot growth of SD vines is retarded or restricted and is known as “restricted spring growth” [12] (Figure 1b). Following véraison (the onset of grape berry ripening and a change from green to red skin in red-berried cultivars), shoots display symptoms of delayed maturity (e.g., lack of lignification), and often exhibit green “islands” on the periderm (Figure 1c) [12]. Over time, the leaves transition to a bright crimson colour with either green veins or red veins (Figure 1d) and persist on the vine into the dormant season, i.e., have delayed senescence. The symptoms of GVA-associated SD have been observed in several grapevine cultivars including Shiraz, Malbec, and Merlot in South Africa and Australia [9,12]. To date, SD has not been reported in other viticultural regions of the world, where GVA is known to be associated with Kober stem grooving [67]. In warmer viticultural regions of Australia (e.g., Riverland, SA), vine decline has also been observed.

### 4.2. Leafroll Disease

**Figure 2:**
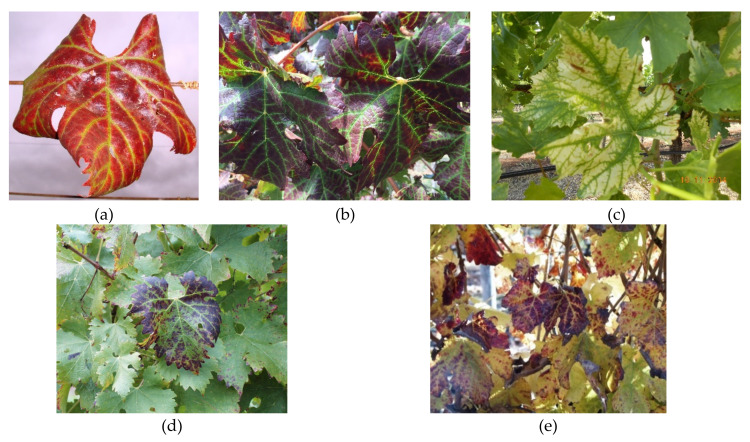
Symptomatology of grapevine leafroll disease. (**a**) leaf margins curled downwards towards the abaxial side of the leaf; (**b**) interveinal regions of leaf blades appear dark red or purple in colour with distinct green veins; (**c**) leaves of white cultivars sometimes appear slightly chlorotic, and the veins may remain green; (**d**) GLRaV-4 strain 9 symptoms on Cabernet Sauvignon; (**e**) GLRaV-4 strain 9 symptoms on Shiraz.

In red-berried cultivars, visible symptoms of grapevine leafroll disease (GLD) typically appear around véraison. However, the virus can be detected using molecular techniques at the pre-véraison stage [68]. Basal leaves turn red, thick and have a marked cupped appearance with the leaf margins curled downwards towards the abaxial side of the leaf (Figure 2a).

Red grape cultivars are more sensitive to GLD and can develop dark red or purple colouration in the interveinal sections of the leaf, often with distinct green veins (Figure 2b), while leaves of white grape cultivars turn yellow or remain symptomless (Figure 2c). Mild symptoms are most observed in grapevines infected with GLRaV-4 and its strains 5, 6, 9, Car, De, and Pr (Ampelovirus, subgroup II). The symptoms associated with GLRaV-4 strain 9 are more severe in Shiraz than in Cabernet Sauvignon, especially at later phenological stages of development (Figure 2d,e). It remains unknown why GLD symptoms develop at specific phenological stages. Naidu et al. [69] suggested that the degree of virus-host interaction may be based on the phenological stage of development resulting from the response of the host’s cellular machinery to the virus.

## 5. High Throughput Sequencing (HTS) and Phylogenetic Analysis of Viruses Associated with Shiraz Disease

### 5.1. High Throughput Sequencing (HTS)

Although some leafroll viruses may produce SD-like symptoms [70], GVA has been proposed as the key pathogen associated with SD [71]. Therefore, an initial metagenomics HTS experiment using the Illumina Miseq platform [72] was conducted in several vineyards known to be infected with GVA with the goal of investigating the virus diversity in symptomatic and non-symptomatic grapevines. In our initial HTS results, we sequenced vines of four Shiraz isolates BV1, BV4, LC1, and LC16 selected from two regions (Table 4). The potential viral agents of SD are highlighted in bold. LC1 represented vines with severe Shiraz disease symptoms, and BV1 represented vines in which GVA and GLRaVs had been previously detected but only showed mild leafroll symptoms. BV4 and LC16 represented symptomless Shiraz vines from the same region. The virus profile for a given cultivar infected with SD was different; however, GVA and grapevine rupestris stem pitting-associated virus (GRSPaV) were always present. The latter, as well as grapevine yellow speckle viroid 1 (GYSVd-1) and hop stunt viroid, was present in both SD-affected and non-SD-affected vines (Table 4).

### 5.2. Phylogenetic Analysis of Viruses Associated with Shiraz Disease

Several full-length and partial sequences of SD associated viruses obtained in this study using HTS are listed in Table 5. A summary of GVA and GLRaV-3 isolates from Australia and their phylogenetic groups is also provided in Table 5.

**Figure 3:**
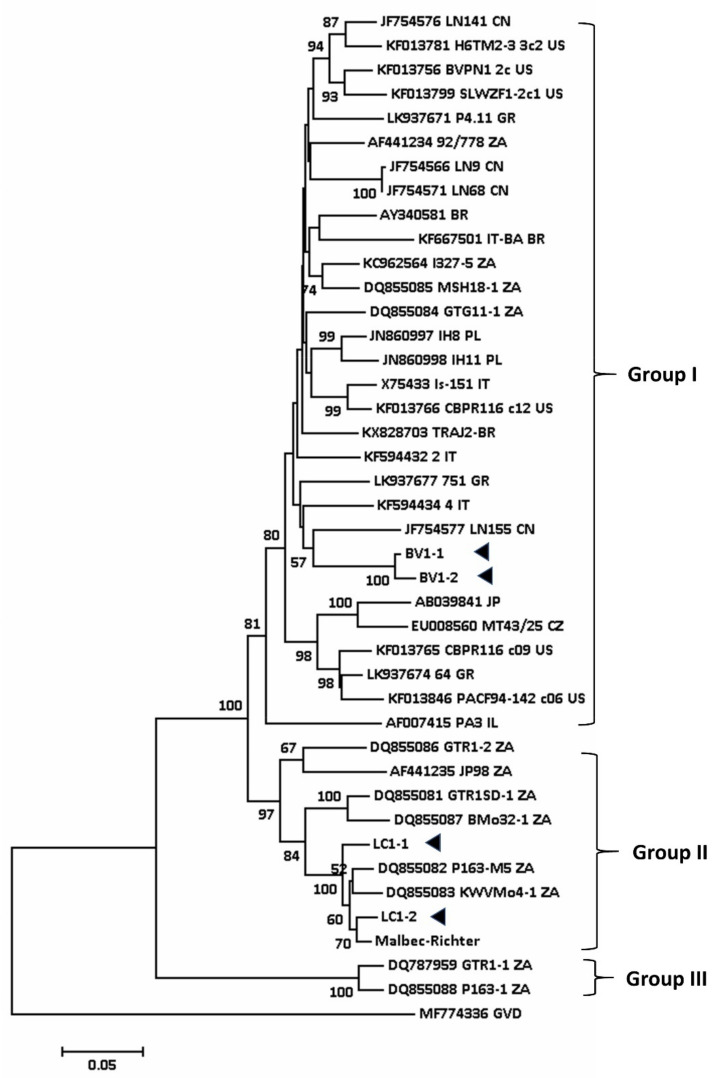
Phylogenetic tree constructed from the alignment of full-length nucleotide sequence of the coat protein of selected isolates of GVA detected in the grapevine using neighbour-joining method (Mega 7) with 1000 bootstrap replications. Bootstrap values less than 50% are not shown. Arrowheads denote the GVA isolates from Australia studied in this work. GVD (MF774336) presents outgroup of this tree.

The sequence of the coat protein gene of the Australian GVA isolates was compared with 40 other isolates available on GenBank, and a phylogenetic tree was constructed (Figure 3). The tree showed that the coat protein (CP) sequences of SD-associated GVA isolates in South Africa (P163-M5, GTR1SD-1) and Australia (LC1-1, LC1-2, Malbec/Richter) are closely related and grouped together (Group II, Figure 3). These isolates are distinct from non-SD GVA isolates BV1-1 and BV1-2 (Group I) [8,73]. BV1-1 and BV1-2 were isolated from old vines (> 150 years old) that have undergone frequent virus testing. Recently, we found symptomless Shiraz grapevines in Clare Valley, SA that tested positive for GVA.

**Figure 4:**
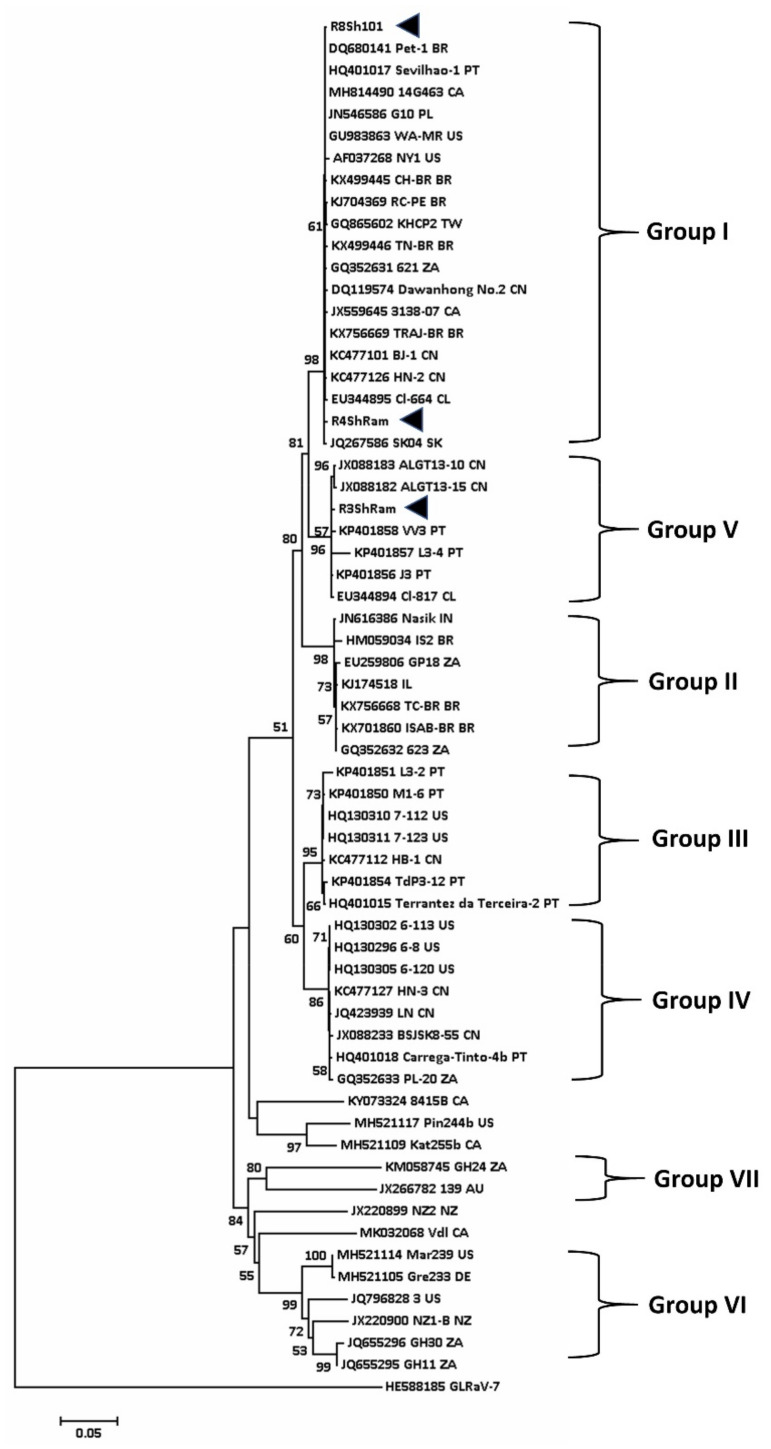
Phylogenetic tree constructed from the alignment of full-length nucleotide sequence of the coat protein of various isolates of GLRaV-3 (see also [73]) detected in the grapevine using Mega 7. A total of 1000 bootstrap replications were performed using neighbour-joining method. Bootstrap values less than 50% are not shown. Arrowheads denote the Australian isolates studied in this work. GLRaV-7 (HE588185) presents outgroup of this tree.

For GLRaV-3, the phylogenetic study was based on nearly complete sequence of the viral CP gene (Figure 4). A total of seven groups were identified. This is slightly different from the grouping of GLRaV-3 isolates reported by Diaz-Lara and co-workers who found 10 phylogenetic groups, but Groups IV and VIII were not depicted in their tree [74]. The Australian isolates are assigned to Groups I, V, and VII (Figure 4 and Table 5).

It is interesting to note that in R3ShRam andR4ShRam that were isolated from the same Shiraz vineyard in Riverland, SA, two different groups of GLRaV-3 (Groups I and IV) were detected (Table 5). Table 5 also shows the sequence of SD-affected Malbec (isolate Malbec-Richter). Based on the CP sequence of GVA and partial sequence of GLRaV-3, the isolate of Malbec-Richter was infected with Group II of GVA and Group I of GLRaV-3 (Table 5; [74]).

Two mild isolates of GLRaV-3 were detected in Australia, both belonging to Group VII. CSL isolated from Crimson Seedless (Accession MT081182) table grapes from Western Australia showed mild leafroll symptoms; this isolate clustered with a symptomless isolate 139 (Figure 4) of the virus detected in cultivar Sauvignon Blanc from the Adelaide Hills, SA (Accession JX266782). Both these isolates are clustered in Group VII, a group that appears to accommodate mild isolates of GLRaV-3. Isolate CSL (13,865 bp, ORF1 to 5) share 99% nucleotide sequence identity with the South African isolate GH24 (Accession KM058745), although only 300 nt of its ORF6 (CP) was sequenced. The truncated CP was not included in Figure 4.

## 6. Virus Effects on Vine Physiology and Fruit Composition

Virus-related diseases can affect grapevine physiological performance, vigour, yield, grape and wine composition, and quality [8,75]. Changes to vine physiology resulting from virus infection primarily relate to photosynthesis and chlorophyll *a* fluorescence, processes that directly or indirectly relate to the vine’s ability to maintain vegetative and reproductive growth as well as ripen crop. Both photosynthesis and chlorophyll fluorescence were observed to be lower in GLRaV-3-infected Cabernet Franc grapevines in a Michigan (USA) vineyard compared to healthy vines after véraison [75]. Following véraison, sugar accumulates in grape berries, and reduced leaf photosynthesis would explain the lower sugar accumulation in the GLRaV-3-infected berries [8]. In the same Cabernet Franc vineyard, reductions in yield and grape soluble solids of 40% and 43%, respectively, were observed compared to uninfected vines. In the Finger Lakes region of New York (USA), Martinson and colleagues reported that grape soluble solids were lower by 2 °Brix in vines affected by GLD [76]. Yield losses have been reported in vines with leafroll disease as well as those associated with the rugose wood complex [7,77,78,79,80,81]. In a study involving several rootstocks, decreased vine vigour and pruning weights were observed in vines infected with GLRaV-1, GLRaV-3, GFLV, and rugose wood associated viruses [82]. In extreme cases, vine death has been observed with infections involving the rugose wood complex [81].

A study on Cabernet Sauvignon grapevines in Chile found that the main anthocyanin and sugar metabolism genes were down-regulated during fruit ripening in GLRaV-3-infected vines [83]. In Pinot Noir in Oregon, reduced total and individual anthocyanin concentrations were observed in GLD vines [80]. It is plausible that the reduced accumulation of grape primary metabolites, e.g., sugars, due to virus infection results in reduced accumulation of secondary metabolites, e.g., anthocyanins; this may be related to decreased phloem loading and hence accumulation of sugars in leaves [69]. Diminished grape composition resulting from changes in aroma compounds such as monoterpenes has been observed with leafroll infections [84].

A preliminary study in Southern Oregon, USA, on Grapevine red blotch virus (GRBV)-infected Cabernet Franc grapevines indicated that virus infections can be detected prior to véraison before the appearance of red leaf symptoms based on measurements of leaf chlorophyll fluorescence (Pagay and Martin, unpublished). Leaf reddening may be a response of grapevines to virus infection in which they accumulate anthocyanins that play a protective role as antioxidants in scavenging free radicals produced by the vine under biotic stress [69]. Nearly all the published studies to date, including those discussed above, have focused on the effects of GLD on red grape cultivars. Much less is known about the effects of GLD on white grape cultivars as well as about the effects of SD on vine physiology and fruit composition of both red and white cultivars. It was observed that GVA-infected Marzemino grapevines had reduced bud fertility, but surprisingly, when co-infected with GLRaV-1, the vines had neither significantly altered yields nor juice soluble solids, total acidity, pH, anthocyanins, or polyphenols [85].

Conversely, some virus species and isolates may not cause disease or impact yield or grape quality particularly when they infect grapevines in the absence of other viruses [86]. Interestingly, some studies suggest that there may be benefits from virus infection in grapevines. “Crimson Seedless” vines inoculated with a mixture of GLRaV-3 (Group VII; isolate CSL-WA), -5, and -9 and GVA were observed to have higher berry weight and lighter berry colour, which was more marketable compared to uninfected grapevines [87]. Similarly, the mildly leafroll-affected cultivar “Emperor” produced larger and crisper berries compared to symptomless clones [88]. These studies highlight some of the positive effects of virus infections on grape composition.

Future studies could also consider investigating virus effects on grape berry development and potentially delayed ripening, which are becoming increasingly important in the context of climate change and regional warming [89,90].

## 7. Economic Impacts of GVA and GLRaV-3

Both GLD and SD are debilitating to vineyards due to economic losses associated with yield reductions, lower grape prices due to inferior fruit quality [25], and vine replacement costs. Unfortunately, there is a dearth of information on the economic impacts of both diseases. Below, we discuss the few published studies on the economic impacts of virus-related grapevine diseases that we are aware of.

In Australia, many grape growers may be complacent in their management of virus infections as they consider the magnitude of the issue insignificant relative to other pathogens in the vineyard including trunk diseases (e.g., Eutypa dieback) and powdery mildew. As a result, virus-infected vines are often ignored. A report on the economic impact of virus and related-infections on grapevines indicated that the reduced profit ranged from AU$34 to $103 ha^−1^ yr^−1^ for prevention and control [91]. In the same study, the economic loss due to viruses was estimated around AU$12 million yr^−1^. Some conscious growers, or those whose economic impact is more significant, opt to rogue and replant the vines and even entire vineyards thereby decreasing the risk of virus spreading to neighbouring vines and vineyards. In Australia, the lifespan of a vineyard infected with SD may not exceed six years and is typically destroyed as it becomes economically unviable. In 2012 in the McLaren Vale and Barossa Valley viticulture regions of South Australia, entire blocks of Shiraz vines grafted on to GVA positive Chardonnay were removed six years after grafting due to lack of productivity (N. Habili, unpublished). It is estimated that the cost of removing virus infected vines and replanting in Australia is around AU$70,000 ha^−1^ [7].

## 8. Management of Shiraz Disease and Grapevine Leafroll Disease in Australian Vineyards

Virus-infected grapevines serve as inoculum for vector-based spreading of viruses in vineyards. Inaction will likely result in the spread of viruses within and between vineyard blocks resulting in larger pools of inoculum, much like fungal diseases that are better known to viticulturists. Atallah and co-workers modelled the spread of disease within and between blocks and found that costs of managing the disease are overestimated when only inter-block spatial dynamics, e.g., virus transmission from neighbouring vineyards via vectors, compared to when the effects of within-block disease spread are additionally considered [92]. Several mitigation strategies can be adopted, if grapevines develop visual symptoms of virus infection, e.g., show restricted spring growth, leaf discoloration post-véraison, or cupping of leaf margins. An integrated approach will undoubtedly be the most effective strategy in dealing with any epidemic. The following three-pronged approach has been shown to be highly effective in controlling viruses in South African and New Zealand vineyards [25]: (i) eliminating potential vectors of grapevine viruses such as mealybugs and scale insects to prevent additional spread of the viruses across vineyard blocks [93]; (ii) rogueing infected vines based on *both* visual symptoms and confirmation with molecular diagnosis; and (iii) replanting with certified plant material from an established grapevine nursery with clean source blocks and good sanitation practices.

Routine scouting of vineyard blocks for both virus symptoms and potential vectors such as mealybugs and scale insects should be done to minimize disruption to vine productivity and reduce economic losses associated with virus-related diseases. A combination of systemic and contact insecticides to control mealybugs and scales, in particular at the early stage in the lifecycle, i.e., first and second instar nymphs, or the use of biological control agents, e.g., parasitoids and mating disruption (pheromone traps), can be effective in minimizing the spread of GVA and GLRaV-3 in vineyards [94]. The use of fungicides such as sulphur applied at high rates in vineyards for the chemical control of powdery mildew (*Erysiphe necator*) may indirectly contribute to increases in virus vector populations via their negative effects on parasitoids [95].

GVA and GLRaV-3 have been detected in and transmitted by, amongst others, grapevine scale insects (*Parthenolecanium persicae*), which are the predominant scale species in Australian vineyards [96]. This scale species has an annual lifecycle (one generation per season). Eggs are typically laid early in the growing season between bud burst and flowering in Australian vineyards. The young crawlers inhabit the underside of leaves moving onto the woody structures of the vine later in the season. Due to the high activity levels (feeding, movement) of juvenile scale insects, it is more critical to control them upon emergence compared to the more sedentary adults. Scale control includes using petroleum-based oil sprays, biological control agents, and broad-spectrum insecticides. Petroleum-based oil sprays are effective in reducing scale populations and have less negative effects on beneficial insects as compared to conventional broad-spectrum insecticides [97].

It is now well-documented that mealybugs are a primary vector of GLRaV-3 [28,98] and GVA [23] (Table 2); hence, mealybug control is of vital importance in any management strategy of viruses in vineyards. Mealybug control in vineyards has been effective using insecticides based on buprofezin or organophosphate chemistries early in the growing season, prior to flowering [99,100]. Removal of bark (bark stripping) prior to the application of sprays will increase the efficacy of contact insecticides due to better penetration into the bark where mealybugs often overwinter. Since crawlers are active and potentially effective in spreading viruses, spraying contact insecticides that coincide with their emergence is an appropriate approach. Insecticide application when more than 15 mealybugs per trap are collected has been recommended in South Africa [101].

As part of any vector management strategy, vineyard hygiene practices including cleaning vineyard equipment and removing dead leaves that can harbor mealybugs and destroying ant nests should be implemented. Observing the patterns of potential virus infection within a block can often provide clues on the source of the virus and mode of spread. Infections at or around points of entry into a vineyard suggest the possible role of humans and/or equipment in aiding the spread of vectors.

Roguing and Replanting

Rogueing and replanting is usually the last resort to deal with any virus infections in a vineyard and less than desirable since it makes vine management more challenging with vines of different ages that also affects the overall quality of the crop [102]. This technique has, however, shown to be highly effective in South Africa where the incidence of GLD in a 41 ha vineyard decreased from 100% (fully infected) to 0.027% over 10 years [98].

In an established vineyard, routine scouting for virus symptoms both early and late in the growing season is critical for effective management of any potential infection. Restricted shoot growth in the spring shortly after bud burst is typical in SD vines (Figure 1b); however, since this symptom can be mistaken for other conditions, appropriate diagnosis is required. GLD-infected vines do not typically display restricted shoot growth and only begin to show symptoms of curling leaf margins and reddening around or shortly after véraison. Vines that show any indications of virus-like infection should be flagged for testing using a molecular assay prior to rogueing. Using a spatial bioeconomic model to determine the optimum rogueing strategy in a leafroll-infected vineyard was recommended to rogue symptomatic vines. Their non-symptomatic neighbours should only be rogued when tested positive for virus. This strategy increased net present value (NPV) by 18-19% compared to no intervention [103].

When establishing a new vineyard, or replanting rogued vines, it is essential to use only clean, certified, and virus-tested planting material as no cure for virus-infected vines currently exists once they are established in a vineyard [93]. In many countries, including Australia, certified planting material is available through established and reputable grapevine nurseries that practice good hygiene, conduct regular virus testing of their source blocks, and routinely scout for viruses and their vectors. Nurseries that use shoot tip cultures to propagate and establish their clean mother blocks are generally reliable sources of virus-free material. Routine virus-testing of mother blocks should also be conducted by vine improvement groups and grapevine nurseries.

## 9. Observations of Shiraz Disease in South Australian Vineyards

Over several seasons, observations of mature (established) grapevines indicated consistent patterns of SD expression in regional vineyards across South Australia, the state with the largest winegrape area in Australia (76,292 ha, approx. 52% of Australian winegrape plantings; National Wine Scan, Wine Australia 2019 https://www.wineaustralia.com/market-insights/national-vineyard-scan-2018). These symptoms vary based on phenological stage, environmental conditions, as well as cultivar. In this section, we provide specific regional examples of SD and the symptoms elicited.

**Figure 5:**
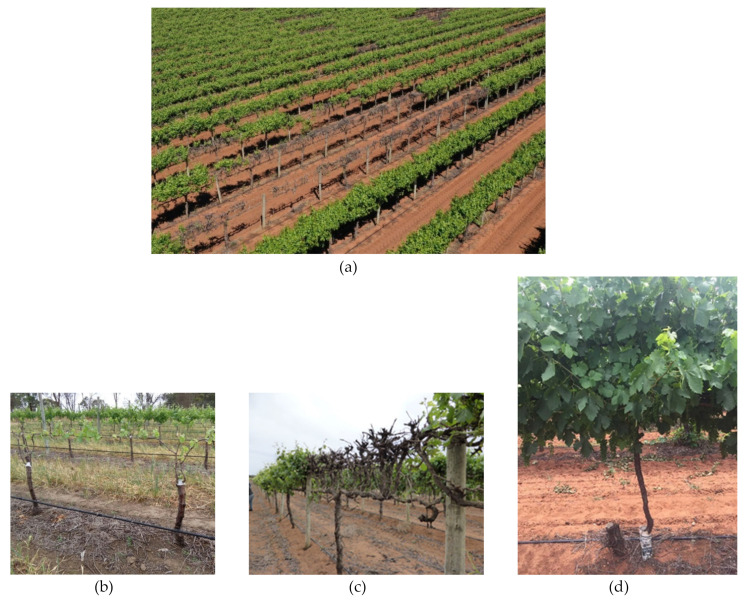
SD-infected grapevines in Australian vineyards. (**a**) Aerial view of a Riverland (South Australia) Shiraz vineyard showing prevalence of Shiraz disease: declining or dead vines with few or no leaves associated with GVA compared to symptomless GVA-infected Chardonnay (two rows on the right); (**b**) Malbec grapevines in a Langhorne Creek (South Australia) vineyard showing typical SD symptoms of restricted spring shoot growth; (**c**) same Shiraz block showing dieback associated with both SD and fungal trunk diseases; (**d**) Shiraz grapevine planted next to a stump, which tested positive for GVA two years later.

Early in the growing season, SD-infected grapevines typically have delayed budburst relative to healthy (non-SD infected grapevines, Figure 5a–c). This delay has been observed to range from several days to one week. Infrequently, in mature blocks where the virus is more established, buds do not burst resulting in a lack of shoot formation and crop and consequent vine decline, as observed in some Riverland vineyards (Figure 5a,c). In the same Riverland vineyard, an uninfected Shiraz grapevine planted next to a GVA-infected stump tested positive two years after planting (Figure 5d).

These vines were subsequently tested and found to be positive for GVA and occasionally GLRaV-3 (Table 4). Vine decline related to SD has only been observed in warmer viticultural regions of South Australia, e.g., Riverland. A complicating factor to the SD story was the discovery of *Diplodia serratia*, a member of *Botryosphaeridae* in the SD affected vines that showed decline in the Riverland, South Australia (Figure 5a). Sequencing of the PCR products using Internal Transcribed Spacer (ITS1, ITS4) primers confirmed the association of this fungus with vine decline. The sequence showed 99% homology with the fungus isolate present in vineyards of southern Spain (accession number: MG745835). Based on this finding, we hypothesise that GVA increases the vulnerability of the vines to fungal pathogen infections. In the same Riverland vineyard (Figure 5a), all the non-SD vines tested positive for *Diplodia* and yet no vine decline was observed, which supports our virus-fungus co-infection hypothesis (N. Habili, unpublished). The cultivar specificity of SD was observed in the same vineyard: adjacent rows of Chardonnay grapevines tested positive for GVA and occasionally for GLRaV-3 but did not show vine decline (Figure 5a, two rows on right). A survey of potential vectors in this “hot spot” of Shiraz and Chardonnay grapevines revealed the presence of mealybugs and grapevine scale that would likely have vectored the viruses.

In vines that had successful budburst, SD infections typically resulted in restricted (or retarded) shoot growth during early stages of vine development. We observed retarded shoot growth symptoms in vineyards in Riverland, McLaren Vale, and Langhorne Creek regions (Figure 5b,c). Shoot growth tends to be retarded when budburst is delayed. This could be a consequence of SD stress-induced reduction in carbohydrate accumulation during bud development resulting from SD. Virus testing of Malbec vines in the Langhorne Creek vineyard confirmed the presence of GVA and GRSPaV. GRSPaV is present in most grapevines worldwide [104] and has been reported to have little or no negative effects on vine productivity [105]. Similar symptoms were observed in an adjacent block planted with Malbec that was previously top-worked onto GVA-infected Chardonnay grapevines confirming that the virus is graft transmissible.

In summary, our observations of SD in Australian vineyards indicate that the disease has the potential to cause vine decline manifested in delayed or no bud burst, retarded shoot development, and low or no crop. SD appears to be cultivar specific, with Shiraz and Malbec showing typical SD symptoms. Other red-berried cultivars such as Gamay, Merlot and Sumoll are also known to show SD symptoms, while Cabernet Sauvignon does not despite specific clones, e.g., SA125 and Reynella, that are infected with GVA.

## 10. Conclusions and Future Work

GVA and GLRaV-3 pose an ongoing threat to Australian vineyards due to their negative consequences on vine vigour, yield, and economic returns to the grower. In a few extreme cases, vineyards experienced yield losses of up to 98%, underscoring the importance of improving our understanding of these viruses; their source; methods of spread; potential vectors and their control; differences in cultivar susceptibility; and, most importantly, the specific interaction between the virus species on grapevine productivity. Systematic research needs to be undertaken to shed light on these open questions and to determine the long-term implications of virus-infected blocks including developing the best strategies to maintain their viability. Advances in proximal and remote sensing technologies provide opportunities for non-destructive detection of virus-infected grapevines [106,107] in a cost-effective manner and on a large spatial scale via airborne platforms [108]. Initial detection using these techniques should be followed by traditional or novel molecular-based testing such as HTS for confirmation of the virus infection. Search for novel viruses, for example, the negative sense RNA viruses of *Bunyavirales* [109], should continue using HTS. Effective virus management in vineyards begins with recognizing virus symptoms, early detection and confirmation, vector control, and rogueing and replanting infected vines with certified material. These management practices have proved effective in several major viticultural regions around the world, and Australian grape growers would be well served to adopt such an approach.

## Figures and Tables

**Table 1:** Common grapevine viruses in Australia, their associated disease, and vectors. Viruses indicated in bold text are the focus of this review.

Family/Genome	Genus	Species ^1^	Associated Disease	Vector
*Betaflexiviridae* Monopartite linear ssRNA (+)	*Vitivirus*	**grapevine virus A** (GVA)	Shiraz Disease, Kober Stem Grooving	Mealybug/scale
		grapevine virus B (GVB)	Corky Bark	Mealybug/scale
	*Foveavirus*	grapevine rupestris stem pitting associated virus (GRSPaV)	Asymptomatic in most, stem pitting	Unknown
	*Trichovirus*	grapevine Pinot gris virus (GPGV)	Leaf mottling and deformation, symptomless	*Colomerus vitis*
*Closteroviridae* Monopartite linear ssRNA (+)	*Ampelovirus*—**Subgroup I**	grapevine leafroll associated virus **(GLRaV)-1** and **GLRaV-3**	Leafroll disease	Mealybug/scale
	*Ampelovirus*—Subgroup II	**GLRaV-4 and its strains: 5, 6, 9 ^2^**	Leafroll disease	Mealybug/scale
	*Closterovirus*	GLRaV-2	Leafroll disease, Graft incompatibility	Unknown
*Secoviridae* Bipartite, linear ssRNA (+)	*Nepovirus*	grapevine fanleaf virus (GFLV) ^3^	Fanleaf, degeneration, decline, chlorosis	*Xiphinema index*; *X. diversicaudatum*
*Tymoviridae* Monopartite, linear ssRNA (+)	*Maculavirus*	grapevine fleck virus (GFkV)	Fleck on *V. rupestris*, Asymptomatic in other *Vitis* sp.	Unknown
	*Marafivirus*	grapevine rupestris vein feathering virus (GRVFV)	Asymptomatic	Unknown

^1^ Viruses associated with Shiraz Disease are highlighted in bold. ^2^ GLRaV-4 strains; Pr^2^, De^2^ and Car^2^ strains have not been detected. ^3^ GFLV has been eradicated [5].

**Table 2:** Common mealybug vectors of grapevine viruses.

Mealybugs	Common Name	Transmitted Viruses	Presence in Australia	References
*Ferrisia gilli* (Gullan)	Gill’s mealybug	GLRaV-3,4	No	[29]
*Heliococcus bohemicus*	Bohemian mealybug	GLRaV-1,3; GVA	No	[30,31,32,33]
*Phenacoccus aceris*	Apple mealybug	GLRaV-1,3,4; GVA; GVB	No	[26,32,34]
*Planococcus citri*	Citrus mealybug	GLRaV-1,3; GVA	Yes	[31,35,36]
*Planococcus ficus*	Grapevine mealybug	GLRaV-1,3, 4; GVA	No	[19,31,37,38]
*Pseudococcus viburni*	Obscure mealybug, tuber mealybug	GLRaV-3; GVA; GVB	Yes	[39]
*Pseudococcus comstocki*	Comstock mealybug	GVE	No	[40]
*Pseudococcus maritimus*	Grape mealybug	GLRaV-1,3	No	[41]
*Pseudococcus calceolariae*	Citrophilus mealybug, scarlet mealybug	GLRaV-3	Yes	[42]
*Pseudococcus longispinus*	Long-tailed mealybug	GLRaV-1,3; GVA	Yes	[22,42,43]

**Table 3:** Common scale insect vectors of grapevine viruses.

Scale Insects	Common Name	Transmitted Viruses	Presence in Australia	References
*Ceroplastes rusci*	Fig wax scale	GLRaV-3,4 strains 5	Yes	[37]
*Coccus hesperidium*	Brown soft scale			[17]
*Coccus longulus*	Long brown scale	GLRaV-3	Yes	[20]
*Parasaissetia nigra*	Nigra scale	GLRaV-3	Yes	[20]
*Parthenolecanium corni*	Brown scale, European fruit lecanium scale	GLRaV-1,3; GVA	Yes	[32,34,48,54,55]
*Parthenolecanium persicae*	Grapevine scale	GLRaV-3; GVA	Yes	[56,57,58,59]
*Parthenolecanium pruinosum*	Frosted scale	Unknown	Yes	[57,58]
Pulvinaria vitis*	Wooly vine scale	GLRaV-3	Yes	[60]
*Pulvinaria innumerabilis*	Wooly maple scale	GLRaV-1,3	No	[61]
Neopulvinaria innumerabilis*	Soft scale	GLRaV-1	Yes	[55]
*Saissetia sp.*	Soft scale	GLRaV-3	Yes	[20]

**Table 4:** Virus profiles in Shiraz Disease (SD)-affected and non-SD-affected Shiraz and Malbec grapevines from South Australia. The potential viral agents of SD are highlighted in bold.

Cultivar	Region	Sampling Year	Sample ID	Isolate Name	SD Symptoms	Viruses Identified	GVA Group
Shiraz	Barossa Valley	2018	BV1	Isolate 1	No	GRSPaV, GRVFV, **GLRaV-1**, **GVA**, GYSVd-1, HSVd	I
Shiraz	Barossa Valley	2018	BV4	Isolate 2	No	GRSPaV, GRVFV, GYSVd-1, HSVd	- ^1^
Shiraz	Langhorne Creek	2018	LC1	Isolate 1	Yes	GRSPaV, **GVA, GLRaV-9,** GRVFV, GYSVd-1, HSV	II
Shiraz	Langhorne Creek	2018	LC16	Isolate 2	No	GRSPaV, GRVFV, GYSVd-1, HSVd	- ^1^
Malbec	Padthaway	2016	Malbec	Malbec-Richter ^2^	Yes	**GVA, GLRaV-3 (4 and its strains 5, 6 & 9),** GRSPaV	II

^1^ No GVA present. ^2^ Malbec on Richter 110 rootstock.

**Table 5:** Accession numbers of the Australian isolates of GVA and GLRaV-3 and their phylogenetic groups studied in this work.

Virus	Variety/Rootstock	Sample ID (Isolate)	Accession#	Sequence Length (bp)	Location	Symptom on Shiraz	Group
GVA	Shiraz	BV1-1	MT070961	2751	Barossa Valley	None	I
GVA	Shiraz	BV1-2	MT070960	597	Barossa Valley	None	I
GVA	Shiraz	LC1-1	MT070963	7363	Langhorne Creek	SD	II
GVA	Shiraz	LC1-2	MT070962	7052	Langhorne Creek	SD	II
GVA	Malbec on Richter	Malbec-Richter	MT070959	598	Padthaway	SD	II
GLRaV-3	Shiraz on Ramsey	R3ShRam	MN984352	942	Riverland	SD	V
GLRaV-3	Shiraz on Ramsey	R4ShRam	MN984353	934	Riverland	SD	I
GLRaV-3	Shiraz on 101-14	R8Sh101	MN984354	941	Riverland	SD	I
GLRaV-3	Malbec on Richter	Malbec-Richter	N/A	N/A	Padthaway	SD	I ^1^

^1^All the contigs of Malbec-Richter matched with the phylogenetic Group I. Malbec-Richter was not depicted in Figure 4 because of a truncated CP sequence.
